# Sugar-sweetened beverages consumption in a multi-ethnic population of middle-aged men and association with sociodemographic variables and obesity

**DOI:** 10.3389/fnut.2022.987048

**Published:** 2022-08-30

**Authors:** Nora A. AlFaris, Naseem M. Alshwaiyat, Hana Alkhalidy, Jozaa Z. AlTamimi, Reham I. Alagal, Reem A. Alsaikan, Malak A. Alsemari, Mona N. BinMowyna, Nora M. AlKehayez

**Affiliations:** ^1^Department of Physical Sports Sciences, College of Education, Princess Nourah bint Abdulrahman University, Riyadh, Saudi Arabia; ^2^School of Nutrition and Dietetics, Faculty of Health Sciences, Universiti Sultan Zainal Abidin, Kuala Nerus, Terengganu, Malaysia; ^3^Department of Nutrition and Food Technology, Faculty of Agriculture, Jordan University of Science and Technology, Irbid, Jordan; ^4^Department of Health Sciences, College of Health and Rehabilitation Sciences, Princess Nourah bint Abdulrahman University, Riyadh, Saudi Arabia; ^5^Department of Medical Imaging – MRI, King Abdullah bin Abdulaziz University Hospital, Princess Nourah bint Abdulrahman University, Riyadh, Saudi Arabia; ^6^College of Applied Medical Sciences, Shaqra University, Shaqra, Saudi Arabia

**Keywords:** sugar-sweetened beverages, multi-ethnic, middle-aged men, sociodemographic, obesity

## Abstract

**Background:**

Adults frequently consume sugar-sweetened beverages. These products are linked to negative health effects such as obesity. Our study was carried out to assess rates of weekly and daily sugar-sweetened beverages consumption in a multi-ethnic population of middle-aged men and association with sociodemographic variables and obesity.

**Methods:**

A sum of 1,800 middle-aged men (36–59 years) living in Riyadh, KSA, participated in this cross-sectional study. Sociodemographic variables and the frequency of sugar-sweetened beverages consumption were gathered from participants using face to face interviews. Weekly and daily consumption of sugar-sweetened beverages were the two binary outcome variables applied in this research. Weight and height were measured following standard procedures.

**Results:**

In this study, 93.8 and 32.6% of participants consumed sugar-sweetened beverages weekly and daily, respectively. The weekly and daily sugar-sweetened beverages consumption was predicted by nationality. Subjects from Pakistan (99.3%) and Yemen (60.0%) reported the greatest rates of weekly and daily consumption, respectively, while Bangladeshi and Sudanese subjects reported the lowest rates of weekly (87%) and daily (2.9%) consumption, respectively. Another factor that predicted weekly sugar-sweetened beverages consumption was obesity. Obese subjects had a significantly greater odds ratio of weekly sugar-sweetened beverages intake than non-obese individuals (OR = 3.80, *P* = 0.003).

**Conclusion:**

Consumption of sugar-sweetened beverages is common among middle-aged men who live in KSA. Results show connecting sugar-sweetened beverages intake with specific sociodemographic variables and obesity.

## Introduction

Globally, a widespread intake of sugar-sweetened beverages (SSBs) has been documented in adults (1). Common SSBs include regular soda, fruit drinks, and energy drinks. Most of SSBs are manufactured by adding high amounts of various types of free sugars such as sucrose and fructose (2). Consumption of SSBs contributes to daily energy intake due to their high added sugar content (3). Thus, SSBs intake may decline overall diet quality and lower daily intake of essential micronutrients (4). Consumption of SSBs is linked to a greater risk of numerous health problems like obesity, diabetes, cardiovascular disease and dental caries (5–7). Subsequently, SSBs intake has been considered an important public health issue (8). This situation motivated the WHO to create guidelines encouraging adults to limit added sugar intake to 5–10% of typical day calories (9). As SSBs consumption is very common among adults, it is vital to investigate patterns of SSBs intake and related sociodemographic variables to create efficient wellbeing promotion initiatives to minimize their use (10, 11).

In the Kingdom of Saudi Arabia (KSA), adult obesity is a serious health issue accompanied by unhealthy lifestyle choices such as physical inactivity and poor diet, including high consumption of SSBs (12–16). Indeed, KSA is the top oil producer in the Middle East region, and its economy is rising quickly. It should be no surprise that KSA hires people from all over the world. In fact, KSA had a workforce of about 50% foreigners, and 90% of the private sector occupations were held by foreigners (17). About one-third of the people in this country are non-Saudi residents, with men making up roughly three-fourths of them (18). Immigrants from various ethnic backgrounds make exploring the disparities in food habits and connections to wellness and illness in a diverse population exciting. Therefore, the objectives of this study were to determine the rates of weekly and daily SSBs consumption in a population of middle-aged men from various ethnic groups and the association with socioeconomic variables and obesity.

## Methods

### Study sample and design

The data for this study were derived from a research project named the Relationship between Obesity, physical Activity, and Dietary pattern among men in KSA (ROAD-KSA) (15, 16, 19–22). It is a cross-sectional research project that took place in Riyadh, KSA, from February to June 2019. The study sample was randomly drawn from Riyadh public areas using a stratified clustered sampling technique. The eligible subjects were middle-aged men aged 36–59 years, living in Riyadh city, without any physical disability (the disability nature may affect body weight and height, which could bias the outcome of obesity diagnosis, and may affect food habits, including SSBs intake which could bias the outcome of SSBs consumption frequency), and having citizenship from one of these countries: KSA, Egypt, Yemen, Syria, Jordan, Sudan, Turkey, Pakistan, Afghanistan, India, Bangladesh, and the Philippines. Before taking part in our study, informed consent was collected from subjects in agreement with the Helsinki Declaration. The present study was authorized by the research ethics committee of Princess Nourah bint Abdulrahman University.

### Sociodemographic data

Professional research team gathered sociodemographic data following face to face interviews. The collected data were nationality, age, residency period in KSA, household type, marital status, educational level, and monthly income.

### Body mass index

Professional research team measured weight and height of subjects while wearing minimal clothes and no slippers in a complete standup situation. Weight was measured to the closest 0.1 kg using a weight balance (Seca 802, Hamburg, Germany), whilst height was measured to the closest 0.1 cm using a portable stadiometer (Seca 213, Hamburg, Germany). Weight (kg) was divided by height (m^2^) to find body mass index (BMI) value. Obesity is determined as a BMI ≥ 30 (23).

### Sugar-sweetened beverages intake

The frequency of SSBs intake was determined using a valid and reliable questionnaire. The questionnaire's face validity was assessed using an objective evaluation from five skilled researchers. A test-retest pilot study with a 14 days' time lag was used to judge the questionnaire's reliability. Data were collected by a skilled research team using face to face interviews. In this study, SSBs were defined as artificial beverages with free sugars that are not recognized as diet or low-calorie, excluding coffee, tea, dairy, and alcoholic beverages (11). Consequently, SSBs were classified into three subtypes: regular soda, fruit drinks and energy drinks. Regular soda refers to carbonated beverages with added sugar. Fruit drinks are artificial fruit juices with free sugars, excluding natural fruit juices. Energy drinks are any carbonated beverage with added sugar and a high level of stimulating ingredients such as caffeine. To determine the frequency of SSBs consumption, the participants were questioned about the number of servings (on average 12 fl oz can or bottle) from each SSBs subtype they usually drink per week or day during the preceding year. The frequency of weekly and daily consumption for total SSBs and each SSBs subtype were computed based on replies. Drinking at least one serving per week was considered weekly consumption, while drinking at least one serving per day was considered daily consumption (24).

### Statistical analysis

Data analysis was managed using Statistical Package for the Social Sciences (SPSS) software for Windows, version 26.0 (SPSS Inc., Armonk, NY, USA). This study uses two binary outcome variables: weekly and daily consumption of SSBs. Categorical variables were analyzed using the Chi-square test, and the results were displayed as frequencies and percentages. After adjusting for studied sociodemographic variables and obesity, a multivariate logistic regression analysis was done to investigate variables linked to weekly and daily SSBs consumption. Two-tailed testing was used to determine *P*-values. Statistical significance was recognized when *P* < 0.05.

## Results

The study subjects came from twelve different countries who lived in KSA. The mean age of study participants was 40.9 ± 3.8 years, and the mean BMI was 26.6 ± 3.6. Table 1 displays the prevalence of weekly SSBs consumption among subjects by sociodemographic variables and obesity. Weekly SSBs consumption was reported in 93.8% of the full study sample, while weekly consumption rates for regular soda, fruit drinks, and energy drinks were 81.9, 58.1, and 32.1%, respectively. When participants were divided into subgroups depending on their nationality, significant differences (*P* < 0.001) in weekly SSBs consumption rates were observed. The participants from Pakistan have the highest rate of SSBs intake per week (99.3%), while the subjects from Bangladesh have the lowest rate (87%). The highest prevalence of regular soda drinking per week (89.8%) was observed among Afghani subjects, while the lowest prevalence (71.8%) was observed among Jordanian subjects. While Filipino subjects have the highest weekly consumption rates of fruit drinks (85.9%) and energy drinks (53.5%). The lowest rates of fruit drinks (24.0%) and energy drinks (17.8%) were reported among subjects from Bangladesh and Sudan, respectively. Participants who have lived in KSA for more than 5 years had a significantly higher prevalence of weekly SSBs intake (94.4%) than those who have been there for 5 years or less (91.3%). Living within non-family households was correlated with a significantly greater rate of weekly SSBs intake (95.8%) than living within family households (90.5%). Single subjects have significantly higher weekly consumption rates of SSBs (95.5%) and regular soda (88.8%) than married participants (93.4 and 81.2%, respectively). A low education level was correlated with a significantly greater weekly SSBs intake rate (95.9%) than high education level (90.0%). Likewise, low monthly income was connected with a significantly greater weekly consumption rate of SSBs (95.9%) than high monthly income (91.6%). Lastly, obese participants have a significantly greater prevalence of weekly SSBs consumption (97.7%) than non-obese subjects (93.2%).

**Table 1:** Prevalence of weekly sugar-sweetened beverages (SSBs) consumption among study participants (*n* = 1,800) stratified by sociodemographic variables and obesity.

**Variables**	**Total SSBs**	**Regular soda**	**Fruit drinks**	**Energy drinks**
All Participants	1,689 (93.8%)	1,475 (81.9%)	1,045 (58.1%)	578 (32.1%)
**Nationality**
Saudi	144 (89.4%)	126 (78.3%)	112 (69.6%)	30 (18.6%)
Egyptian	153 (95.0%)	138 (85.7%)	123 (76.4%)	47 (29.2%)
Yemeni	114 (99.1%)	97 (84.3%)	97 (84.3%)	48 (41.7%)
Syrian	141 (89.8%)	118 (75.2%)	110 (70.1%)	65 (41.4%)
Jordanian	148 (87.1%)	122 (71.8%)	125 (73.5%)	40 (23.5%)
Sudanese	162 (93.1%)	140 (80.5%)	49 (28.2%)	31 (17.8%)
Turkish	237 (96.0%)	208 (84.2%)	112 (45.3%)	87 (35.2%)
Pakistani	143 (99.3%)	129 (89.6%)	75 (52.1%)	46 (31.9%)
Afghan	145 (98.6%)	132 (89.8%)	75 (51.0%)	65 (44.2%)
Indian	147 (96.1%)	124 (81.0%)	82 (53.6%)	51 (33.3%)
Bangladeshi	87 (87.0%)	84 (84.0%)	24 (24.0%)	30 (30.0%)
Filipino	67 (94.4%)	57 (80.3%)	61 (85.9%)	38 (53.5%)
*P*-value^*^	**<0.001**	**<0.001**	**<0.001**	**<0.001**
**Residency Period in KSA**
1–5 years	303 (91.3%)	259 (78.0%)	198 (59.6%)	105 (31.6%)
6 years or more	1,386 (94.4%)	1,216 (82.8%)	847 (57.7%)	473 (32.2%)
*P*-value^*^	**0.031**	**0.039**	0.517	0.834
**Household Type**
Non-family household	1,088 (95.8%)	969 (85.3%)	572 (50.4%)	410 (36.1%)
Family household	601 (90.5%)	506 (76.2%)	473 (71.2%)	168 (25.3%)
*P*-value^*^	**<0.001**	**<0.001**	**<0.001**	**<0.001**
**Marital status**
Single	208 (97.2%)	187 (87.4%)	126 (58.9%)	69 (32.2%)
Married	1,481 (93.4%)	1,288 (81.2%)	919 (57.9%)	509 (32.1%)
*P*-value^*^	**0.029**	**0.028**	0.795	0.965
**Education level**
Low (high school or less)	1,133 (95.9%)	1,005 (85.0%)	596 (50.4%)	394 (33.3%)
High (college or more)	556 (90.0%)	470 (76.1%)	449 (72.7%)	184 (29.8%)
*P*-value^*^	**<0.001**	**<0.001**	**<0.001**	**<0.001**
**Monthly income**
Low (<1,000 USD)	954 (95.6%)	838 (84.0%)	535 (53.6%)	359 (36.0%)
High (≥1,000 USD)	735 (91.6%)	637 (79.4%)	510 (63.6%)	219 (27.3%)
*P*-value^*^	**0.001**	**0.013**	**<0.001**	**<0.001**
**Obesity (BMI** **≥30)**
No	1,431 (93.2%)	1,257 (81.8%)	887 (57.7%)	513 (33.4%)
Yes	258 (97.7%)	218 (82.6%)	158 (59.8%)	65 (24.6%)
*P*-value^*^	**0.004**	0.773	0.523	**0.005**

* Categorical variables were analyzed by using Chi-squared test and expressed as numbers and percentages. Significant values (*P*-value < 0.05) were presented in Bold type.

Table 2 shows the prevalence of daily SSBs consumption among study participants by sociodemographic variables and obesity. Daily consumption of SSBs was reported in 32.6% of the full study sample, whereas the rates for regular soda, fruit drinks, and energy drinks were 11.7, 2.6, and 0.8%, respectively. When participants are subdivided depending on their nationality, significant variations (*P* < 0.001) in daily intake rates of SSBs, regular soda and fruit drinks are identified. Yemeni subjects have the highest rates of daily SSBs intake (60.0%) and regular soda (20.9%). In comparison, the highest rates of daily intake of fruit drinks and energy drinks were seen among Filipino (12.7%) and Afghan (2.7%) subjects, respectively. In contrast, the lowest rates of daily consumption of SSBs (2.9%) and regular soda (0.6%) were reported among Sudanese and Jordanian subjects, respectively. However, daily consumption of fruit drinks was not observed among Sudanese, Pakistani, Indian, and Bangladeshi subjects, while daily consumption of energy drinks was not observed among Egyptian, Jordanian, Sudanese, and Bangladeshi subjects. The daily SSBs consumption rates among participants with 5 years or less and more than 5 years of residency in KSA were 36.4 and 31.7%, respectively (*P* > 0.05). Similarly, the daily SSBs consumption rates among participants who live within non-family and family households were 33.0 and 31.9%, respectively. The daily SSBs consumption rates among single and married participants were 37.4 and 32.0%, respectively. The daily SSBs consumption rates among participants with low and high education levels were 32.3 and 33.2%, respectively. The daily SSBs consumption rates among participants with low and high monthly income were 32.0 and 33.4%, respectively. Finally, the daily SSBs consumption rates among obese and non-obese participants were 28.8 and 33.3%, respectively.

**Table 2:** Prevalence of daily sugar-sweetened beverages (SSBs) consumption among study participants (*n* = 1,800) stratified by sociodemographic variables and obesity.

**Variables**	**Total SSBs**	**Regular soda**	**Fruit drinks**	**Energy drinks**
All participants	587 (32.6%)	210 (11.7%)	46 (2.6%)	14 (0.8%)
**Nationality**
Saudi	41 (25.5%)	14 (8.7%)	2 (1.2%)	1 (0.6%)
Egyptian	96 (59.6%)	33 (20.5%)	10 (6.2%)	0 (0.0%)
Yemeni	69 (60.0%)	24 (20.9%)	10 (8.7%)	3 (2.6%)
Syrian	58 (36.9%)	20 (12.7%)	3 (1.9%)	2 (1.3%)
Jordanian	49 (28.8%)	1 (0.6%)	6 (3.5%)	0 (0.0%)
Sudanese	5 (2.9%)	5 (2.9%)	0 (0.0%)	0 (0.0%)
Turkish	75 (30.4%)	50 (20.2%)	4 (1.6%)	1 (0.4%)
Pakistani	40 (27.8%)	7 (4.9%)	0 (0.0%)	1 (0.7%)
Afghan	61 (41.5%)	23 (15.6%)	2 (1.4%)	4 (2.7%)
Indian	45 (29.4%)	27 (17.6%)	0 (0.0%)	1 (0.7%)
Bangladeshi	11 (11.0%)	2 (2.0%)	0 (0.0%)	0 (0.0%)
Filipino	37 (52.1%)	4 (5.6%)	9 (12.7%)	1 (1.4%)
*P*-value^*^	**<0.001**	**<0.001**	**<0.001**	0.074
**Residency Period in KSA**
1–5 years	121 (36.4%)	49 (14.8%)	13 (3.9%)	1 (0.3%)
6 years or more	466 (31.7%)	161 (11.0%)	33 (2.2%)	13 (0.9%)
*P*-value^*^	0.099	0.052	0.082	0.274
**Household type**
Non-family household	375 (33.0%)	168 (14.8%)	24 (2.1%)	10 (0.9%)
Family household	212 (31.9%)	42 (6.3%)	22 (3.3%)	4 (0.6%)
*P*-value^*^	0.636	**<0.001**	0.119	0.517
**Marital status**
Single	80 (37.4%)	38 (17.8%)	9 (4.2%)	1 (0.5%)
Married	507 (32.0%)	172 (10.8%)	37 (2.3%)	13 (0.8%)
*P*-value^*^	0.113	**0.003**	0.103	0.582
**Education level**
Low (high school or less)	382 (32.3%)	170 (14.4%)	19 (1.6%)	8 (0.7%)
High (college or more)	205 (33.2%)	40 (6.5%)	27 (4.4%)	6 (1.0%)
*P*-value^*^	0.714	**<0.001**	**<0.001**	0.5
**Monthly income**
Low (<1,000 USD)	319 (32.0%)	124 (12.4%)	29 (2.9%)	7 (0.7%)
High (≥1,000 USD)	268 (33.4%)	86 (10.7%)	17 (2.1%)	7 (0.9%)
*P*-value^*^	0.514	0.264	0.294	0.681
**Obesity (BMI** **≥30)**
No	511 (33.3%)	179 (11.7%)	38 (2.5%)	12 (0.8%)
Yes	76 (28.8%)	31 (11.7%)	8 (3.0%)	2 (0.8%)
*P*-value^*^	0.151	0.967	0.597	0.968

* Categorical variables were analyzed by using Chi-squared test and expressed as numbers and percentages. Significant values (*P*-value < 0.05) were presented in Bold type.

Table 3 demonstrates the odds ratios for weekly and daily SSBs consumption among participants for sociodemographic variables and obesity. Nationality was found to be a predictor of weekly and daily SSBs consumption. Compared to Bangladeshi participants, subjects from Egypt, Yemen, Turkey, Pakistan, Afghanistan, and India had significantly greater odds ratios of SSBs consumption per week [odds ratio (OR) ranged from 2.96 to 29.19, *P* < 0.05]. Likewise, compared to subjects from Sudan, subjects from the remaining countries had significantly greater odds ratios of daily SSBs consumption (OR ranging from 3.72 to 103.15, *P* < 0.05). Furthermore, increasing age was significantly associated with lower odds ratios of weekly (OR = 0.88, *P* < 0.001) and daily (OR = 0.95, *P* < 0.001) SSBs consumption. Subjects who lived in family households had a significantly lower odds ratio of daily SSBs consumption than those who lived in non-family households (OR = 0.41, *P* < 0.001). Highly educated subjects had a significantly lower odds ratio of daily SSBs consumption than those with low education (OR = 0.60, *P* = 0.006). Subjects with a high monthly income had a significantly higher odds ratio of daily SSBs consumption than those with low monthly income (OR = 1.35, *P* = 0.044). Finally, obese subjects had a significantly higher odds ratio of weekly SSBs consumption than non-obese subjects (OR = 3.80, *P* = 0.003).

**Table 3:** Odds ratios of weekly and daily sugar-sweetened beverages (SSBs) consumption among study participants for sociodemographic variables and obesity.

**Variables**	**Weekly SSBs consumption**		**Daily SSBs consumption**	
	Odds ratio (95% CI)^*^	***P*-value**	Odds ratio (95% CI)^*^	***P*-value**
**Nationality**
Saudi	2.40 (0.79–7.30)	0.122	31.41 (11.29–87.38)	**<0.001**
Egyptian	3.35 (1.12–9.99)	**0.03**	89.76 (33.83–238.19)	**<0.001**
Yemeni	29.19 (3.38–252.28)	**0.002**	103.15 (37.58–283.17)	**<0.001**
Syrian	2.39 (0.81–7.02)	0.113	47.94 (17.55–130.92)	**<0.001**
Jordanian	1.94 (0.68–5.57)	0.216	28.50 (10.37–78.31)	**<0.001**
Sudanese	1.73 (0.71–4.22)	0.226	1	
Turkish	3.81 (1.40–10.36)	**0.009**	12.52 (4.85–32.35)	**<0.001**
Pakistani	16.40 (2.00–134.20)	**0.009**	14.69 (5.54–38.94)	**<0.001**
Afghan	9.81 (2.05–46.93)	**0.004**	25.19 (9.66–65.69)	**<0.001**
Indian	2.96 (1.05–8.38)	**0.041**	12.59 (4.83–32.81)	**<0.001**
Bangladeshi	1		3.72 (1.25–11.11)	**0.019**
Filipino	3.14 (0.84–11.76)	0.09	57.05 (20.13–161.68)	**<0.001**
**Age (years)**	0.88 (0.84–0.93)	**<0.001**	0.95 (0.92–0.98)	**<0.001**
**Residency period in KSA**
1–5 years	1		1	
6 years or more	1.33 (0.79–2.22)	0.283	0.94 (0.70–1.26)	0.665
**Household type**
Non-family household	1		1	
Family household	0.66 (0.30–1.48)	0.314	0.41 (0.28–0.61)	**<0.001**
**Marital status**
Single	1		1	
Married	1.11 (0.44–2.82)	0.82	1.35 (0.94–1.93)	0.102
**Education level**
Low (high school or less)	1		1	
High (college or more)	0.67 (0.32–1.38)	0.274	0.60 (0.42–0.86)	**0.006**
**Monthly income**
Low (<1,000 USD)	1		1	
High (≥1,000 USD)	0.81 (0.42–1.57)	0.527	1.35 (1.01–1.80)	**0.044**
**Obesity (BMI** **≥30)**
No	1		1	
Yes	3.80 (1.60–9.05)	**0.003**	0.72 (0.52–1.00)	0.052

* Multivariate logistic regression analysis was used after adjusting for subjects' sociodemographic variables and obesity. Differences were considered statistically significant at *P*-value < 0.05, and significant values were presented in Bold type.

## Discussion

This study explored weekly and daily SSBs consumption rates in a multi-ethnic population of middle-aged men. Our results disclosed that most subjects (93.8%) were weekly consumers of SSBs and about one-third (32.6%) were daily consumers of SSBs. The prevalence of SSBs consumption among adults has been investigated in several studies. According to a recent nationally representative survey from KSA, 71.2% of adults consumed SSBs weekly, while 35.5% consumed SSBs daily. In addition, 65% of middle-aged adults (35–54 years) were weekly SSBs consumers (24). In a national survey of Australian adults, 55.9 and 19.3% of men consumed SSBs weekly and daily, respectively. The rates of weekly and daily SSBs intake in adults aged 31–45 years were 54.7% and 16.3, respectively, while the rates of weekly and daily SSBs intake in adults aged 46–60 years were 41.1 and 12.5%, respectively (10). A Norwegian study reported that SSBs intake rate among adults was 34% (25). Another study indicated that 63.9% of American adults drank SSBs daily. Moreover, the daily consumption rates of regular soda, fruit drinks, and energy/sport drinks were 21, 6.6, and 5.7%, respectively (26). The rate of daily SSBs consumption among British adults was 20.4% (27). Finally, research from New York City reported that 27.5% of adults were daily consumers of SSBs (28).

This study's data showed that participants from different countries consumed SSBs at significantly different rates. This outcome is in line with several earlier research that found a substantial difference in SSBs intake across adults from various countries, regions, or ethnic origins (26, 29, 30). A study that compared the consumption of SSBs across 187 countries spread over 21 global geographical areas discovered significant variation in SSBs consumption. The difference between the top and lowest regional consumption levels was over ten times. East Asia had the lowest intake of SSBs, while the Caribbean had the highest (30). The consumption of SSBs and SSBs subtypes in the United States differed according to where people lived. Adults in the Northeast, South, West, and Midwest consumed daily SSBs at rates of 68.4, 66.7, 61.2, and 58.8%, respectively. According to findings from this study, adults in the Northeast were much more likely (OR = 1.13) to report consuming SSBs on a daily basis than adults in the South, while those in the Midwest (OR = 0.70) and the West (OR = 0.78) were noticeably less likely to do so (26). In another American study, SSBs intake varies depending on ethnicity. Blacks and Hispanic middle-aged and older adults were more likely than Whites to drink SSBs (29). The causes of these differences in SSBs consumption rates by nationality are still unconfirmed. The potential explanations include differences in the ecological factors such as availability and accessibility to SSBs that adults experienced in their original countries during the preceding age stages (31, 32). Other reasons include variations in how they reacted to SSBs marketing advertisements in the country where they lived caused by disparities in language and cultural standards (10, 33). Nevertheless, when initiatives to lower SSBs consumption are developed, disparities in SSBs consumption rates by nationality should be taken into account (34).

Linking SSBs consumption among adults to sociodemographic factors is widely discussed in previous studies (27, 29). Our results observed significant relationships between weekly or daily SSBs consumption and age, household type, education level and monthly income. Our findings agreed with findings from previous studies that reported a higher likelihood of SSBs consumption was linked to younger age (10, 24, 28, 35, 36), living within non-family households (37), and lower education levels (27, 29). Contrarily, our findings conflict with an earlier study which stated that SSBs consumption did not seem to differ between university students living in their family households and those living in non-family households (38), and earlier studies reported that a higher likelihood of SSBs consumption was linked to lower monthly income (27, 29).

Interestingly, our findings supported the link between consuming SSBs every week and obesity. Many adults are affected by obesity, a costly condition with high morbidity and mortality rates (39, 40). Numerous studies support the relationship between SSBs consumption and adulthood obesity and other obesity-related conditions like diabetes and coronary heart disease (6, 41, 42). Several theories have been established to elucidate the relationship between SSBs use and obesity (43). Adults who usually drink SSBs consume significantly more calories, which causes a positive energy balance (44, 45). On any given day, around 50% of United States adults have one or more SSBs servings, and nearly 7% of their daily caloric intake is derived from SSBs (46). Possible mechanisms include decreased satiety, fast fructose absorption, and after-meal hyperglycemia. The absorption of glucose can cause after-meal hyperglycemia when SSBs are drunk, which reduces the carbohydrate-related elevation in serum glucose level (47). Postprandial hyperglycemia causes a cascade of changes, including hyperinsulinemia, glucose uptake by insulin-sensitive tissues, concurrent hypoglycemia to levels lower than fast values, greater appetite driven by the lack of available fuel, and overeating (48). Furthermore, fructose is processed largely in the liver. Fast fructose absorption from SSBs may overload hepatic metabolic processes, promote lipogenesis, and result in physiological abnormalities such as insulin resistance and excessive fat accumulation (49). Fructose consumption switch on hepatic *de novo* lipogenesis through stimulating two master transcriptional regulators: SREBP-1c and ChREBP. As this process is independent of insulin secretion, fructose consumption can encourage hepatic lipid buildup even in the presence of insulin resistance. Furthermore, fructose intake causes ATP depletion and suppresses the oxidation of fatty acids in the hepatocytes' mitochondria, promoting fat accumulation in the liver and secreting triglycerides-rich VLDL particles. Overall, fructose intake promotes dyslipidemia, cardiometabolic risk factors and insulin resistance, and elevates visceral fat deposit in overweight and obese adults (50, 51).

Several environmental and governmental modifications need to be made to lower SSBs use in the community (11). Limiting the availability of these foods in public places and reducing exposure to SSBs commercial advertisements in the media may help reduce SSBs consumption (10). Moreover, raising public knowledge of the harmful health effects of SSBs use through public health education initiatives may aid efforts to restrict overall SSBs intake (8). In addition, campaigns that encourage substituting SSBs with energy-free liquids like water should be considered (52). Global public health organizations have encouraged governments to intervene due to the widespread and fast increases in SSBs intake. Taxing the price of SSBs is one practical solution to decrease SSBs intake (53, 54).

Few limitations apply to our study. The significant associations between outcome measures and their predictors could not be used to infer causality because of the cross-sectional design. Moreover, women were not involved in the present study. Another drawback was using a self-reported frequency approach to collect consumption data, which relied on participants' memories. Compared to evaluating the consumption using a 24-h recall method, this approach can result in an underestimated SSBs intake. The definition of SSBs is diverse across studies limiting the opportunity to compare our results with previous studies. Finally, this study could not account for calorie intake when analyzing the relationship between SSBs consumption and obesity due to a lack of energy intake data. Despite these limitations, this study still presents valuable data about the rates of SSBs consumption and associated determinants.

## Conclusions

According to this study, middle-aged men who live in KSA consumed SSBs at relatively high rates. The results demonstrated that middle-aged men from different countries who reside in KSA drink SSBs at noticeably varied rates. The findings also show a connection between SSBs consumption, certain sociodemographic variables, and obesity.

## Data availability statement

The raw data supporting the conclusions of this article will be made available by the authors, without undue reservation.

## Ethics statement

The studies involving human participants were reviewed and approved by Research Ethics Committee at Princess Nourah bint Abdulrahman University, Riyadh, Saudi Arabia. The patients/participants provided their written informed consent to participate in this study.

## Author contributions

RIA, RAA, NAls, and NAlF: conceptualization. MA, HA, and JA: methodology. NAlK, RIA, and MA: software. MA and MB: validation. HA and NAls: formal analysis. RAA and NAlK: investigation. NAlF and MB: resources. RAA, HA, and NAls: data curation. NAlF, NAlK, JA, and RIA: writing—original draft preparation. RAA, MB, NAls, and HA: writing—review and editing. JA, MA, and MB: visualization. NAlF: supervision. JA: project administration. RIA and NAlK: funding acquisition. All authors have read and agreed to the published version of the manuscript.

## Funding

This research was funded by Princess Nourah bint Abdulrahman University Researchers Supporting Project number (PNURSP2022R131), Princess Nourah bint Abdulrahman University, Riyadh, Saudi Arabia.

## Conflict of interest

The authors declare that the research was conducted in the absence of any commercial or financial relationships that could be construed as a potential conflict of interest.

## Publisher's note

All claims expressed in this article are solely those of the authors and do not necessarily represent those of their affiliated organizations, or those of the publisher, the editors and the reviewers. Any product that may be evaluated in this article, or claim that may be made by its manufacturer, is not guaranteed or endorsed by the publisher.

## Abbreviations

KSA: Kingdom of Saudi Arabia
BMI: Body Mass index
OR: odds ratio.
